# Selecting age-related functional characteristics in the human gut microbiome

**DOI:** 10.1186/2049-2618-1-2

**Published:** 2013-01-09

**Authors:** Yemin Lan, Andres Kriete, Gail L Rosen

**Affiliations:** 1 School of Biomedical Engineering, Science, and Health Systems, Drexel University, 3141 Chestnut Street, Philadelphia, PA 19104, USA; 2 Department of Electrical and Computer Engineering, Drexel University, 3141 Chestnut Street, Philadelphia, PA 19104, USA

**Keywords:** Metagenomics, KEGG, Pfam, SVM, Supervised classification

## Abstract

**Background:**

Human gut microbial functions are often associated with various diseases and host physiologies. Aging, a less explored factor, is also suspected to affect or be affected by microbiome alterations. By combining functional feature selection with supervised classification, we aim to facilitate identification of age-related functional characteristics in metagenomes from several human gut microbiome studies (MetaHIT, MicroAge, MicroObes, Kurokawa *et al.*’s and Gill *et al.*’s dataset).

**Results:**

We apply two feature selection methods, term frequency-inverse document frequency (TF-iDF) and minimum-redundancy maximum-relevancy (mRMR), to identify functional signatures that differentiate metagenomes by age. After features are reduced, we use a support vector machine (SVM) to predict host age of new metagenomes. Functional features are from protein families (Pfams), Kyoto Encyclopedia of Genes and Genomes (KEGG) pathways, KEGG ontologies and the Gene Ontology (GO) database. Initial investigations demonstrate that ordination of the functional principal components shows great overlap between different age groups. However, when feature selection is applied, mRMR tightens the ordination cluster for each age group, and TF-iDF offers better linear separation. Both TF-iDF and mRMR were used in conjunction with a SVM classifier and achieved areas under receiver operating characteristic curves (AUCs) 10 to 15% above chance to classify individuals above/below mid-ages (about 38 to 43 years old) using Pfams. Better performance around mid-ages is also observed when using other functional categories and age-balanced dataset. We also identified some age-related Pfams that improved age discrimination at age 65 with another feature selection method called LEfSe, on an age-balanced dataset. The selected functional characteristics identify a broad range of age-relevant metabolisms, such as reduced vitamin B12 synthesis, reduced activity of reductases, increased DNA damage, occurrences of stress responses and immune system compromise, and upregulated glycosyltransferases in the aging population.

**Conclusions:**

Feature selection can yield biologically meaningful results when used in conjunction with classification, and makes age classification of new human gut metagenomes feasible. While we demonstrate the promise of this approach, the data-dependent prediction performance could be further improved. We hypothesize that while the Qin *et al.* dataset is the most comprehensive to date, even deeper sampling is needed to better characterize and predict the microbiomes’ functional content.

## Background

The microbial world is vast and diverse. Inside the human body alone, microbes comprise 10 times the total number of human cells and 100 times the total size of human genome [1]. Most of these microbes participate in metabolic activities that interact with the habitat they are growing in with processes such as biodegradation, nitrogen fixation, oxygenic photosynthesis, nutrient production and activation of host immune systems [2]. As a result, microbial structure and function are often associated with a variety of environmental characteristics and host physiologies, such as inflammatory bowel disease (IBD) [3-6], obesity [7,8], nationality [9,10] and diet [11]. There is also interest in studying the association between gut microbiome and human age, even though age is a fuzzy variable compared with more precise quantifications such as disease diagnosis or diet types, since people age at different rates [12]. While the differences of microbiomes between one- to three-year-old children and adults [10,13] and adults and the elderly [14,15] have been studied, the effect of aging on the microbiome has been recognized as a difficult problem [4,10,16,17]. In this paper, we demonstrate that age-related functional characteristics can be identified for metagenomic samples by using feature selection in conjunction with supervised classification.

In the past few years, the correlation between microbial composition and their hosts’ physiology has arisen as a major concern of comparative metagenomics [18]. Ley and Turnbaugh’s study [7] was one of the first to establish the correlation between adiposity and gut microbial ecology, using the relative abundances of two predominant bacterial phyla in human gut. Various dominant microbial species groups in different gastrointestinal tract regions were reported thereafter [19]. Kurokawa *et al.*[13] and Biagi *et al.*[14] have separately shown that human gut microbiomes were capable of discriminating hosts with extreme age ranges. In addition, Qin *et al.*[3] differentiated IBD patients and healthy people with the abundances of their gut bacterial species. More recently, diet was identified as a driving force that shaped gut microbiomes across mammalian phylogeny and among human beings [20].

Functional metagenomic analysis has added a new method to comparing microbiomes. Significantly enriched metabolisms were first reported in two American gut metagenomes, using the KEGG (Kyoto Encyclopedia of Genes and Genomes) pathways and the COGs (Clusters of Orthologous Groups of proteins) [21]. Kurokawa *et al.*[13] studied assignment of additional human metagenomes combined with several environmental metagenomes, and successfully identified metagenomes from children, adults, infants and environments. Both viral genomes and bacterial genomes showed that metabolic pathway profiles could strongly discriminate different natural environments [22]. And similar clustering patterns between the mammalian gut bacterial lineages and gene content further showed that microbiome function correlated to host diets [20]. More recently, Greenblum *et al.*[23] identified both gene-level and enzyme-network-level differences associated with obesity and IBD in the Qin *et al.* human gut microbiome dataset.

Despite an increase in associations between human gut microbial functions and host physiologies, little is known about the age-related microbial activities involved in the development and progression of the human microbiota [16,21,24]. While more and more microbial species are linked to particular characteristics [25], few studies show prediction of novel metagenomic samples using the gene content [22,26], even though there are a variety of ways to identify open reading frames and genes [27,28]. Since the consortia of microbiota is under conjoint influences of various forces, it is difficult to extract the effect caused by aging or to predict the host age based on microbial gene content [29,30].

## Methods

The age classification of unknown metagenomic samples was completed by combining feature selection methods (term frequency-inverse document frequency (TF-iDF) and minimum-redundancy maximum-relevancy (mRMR), described in detail below) to identify functional signatures that best differentiate the samples and a support vector machine (SVM) as the classifier. The functional features were assigned to protein sequences using different databases: the Pfam database (a large collection of protein families), the KEGG database (including KEGG pathways and KEGG ontologies), and the Gene Ontology database (a controlled vocabulary of terms describing gene product characteristics). Two different datasets (the Qin *et al.* dataset and an age-balanced dataset) were used here to examine the classification. The implementation of the following procedures was developed in MATLAB [31] and R [32].

### The Qin *et al*. dataset

We acquired the human gut microbiome data established by Qin *et al.*[3], which is one of the most comprehensive human microbiome datasets to date. The dataset contains metagenomic samples from 124 individuals in Spain and Denmark with various physical conditions. Non-redundant protein sequences for each gut metagenomic sample were acquired from the dataset as well as the annotations for KEGG pathway and KEGG Ontology. Of the 124 individuals, there are 25 IBD patients and 42 obese individuals (where obesity is defined as a Body Mass Index greater than 30, and three of the obese individuals also have IBD). A majority of the subjects are between 40 and 60 years old: six individuals are younger than 30, eight individuals are in their 30s, 39 individuals are in their 40s, 43 individuals are in their 50s and the rest, 28 individuals, are in their 60s.

### Age-balanced dataset

In the Qin *et al.* dataset, over 85% of the samples are from individuals over 40. To examine whether our observation was consistent with population that has a more uniform distribution across age, we constructed an ‘age-balanced’ dataset by combining 52 human gut microbiome samples from multiple studies, including seven Japanese samples [13], two American samples [21], three French samples and six Italian samples [4], as well as 20 samples from Spain and 14 samples from Denmark in the Qin *et al.* dataset. The ages of these samples range from 22 to 87 years old, with six people in their 20s, ten people in their 30s, 40s, 50s and 60s respectively, and three people in their 70s and 80s separately. Additional file 1: Table S1 shows the detailed breakdown of the demographics of the age-balanced dataset.

### Functional annotation

The assignment of Pfam [33] to protein sequences was implemented in the HMMER 3.0 package [34]. A sequence may have no or multiple matches in the Pfam database, above the default Pfam gathering threshold (GA) cutoff value. The number of non-redundant protein sequences assigned to each Pfam was recorded for each metagenomic sample. It should be noted that the number of matching sequences to a Pfam does not imply the abundance or expression level of the corresponding genes; instead, for non-redundant proteins in a metagenome, it implies the diversity of different proteins/genes within one protein family.

For the age-balanced dataset, the non-redundant protein sequences in each metagenome were also aligned using the Basic Local Alignment Search Tool (BLAST) [35] against the Universal Protein Resource (UniProt) databases [36], and the Gene Ontology (GO) [37] terms assigned to each protein sequence were then transferred from its best match (e-value > 10e-5). This is similar to how the MEtaGenome ANalyzer (MEGAN) matches DNA sequences to KEGG ontologies and thus KEGG pathways [38]. The abundances of KEGG pathways and KEGG Ontology identifiers in this paper were acquired directly from the Qin *et al.* dataset.

### Feature selection

The alignment against functional databases gave rise to thousands of unique functional features, hence, it is necessary to extract only features that best describe the differences between metagenomes (in our case the differences occurred along age). We used two feature selection methods to extract the most representative functional signatures for age discrimination, and examined their ability to characterize new samples using a support vector machine (SVM). We also benchmarked a recently proposed feature selection method for age detection.

***TF-iDF*** (term frequency-inverse document frequency) is a probability-based weighting method often used in determining the importance of features in information retrieval and text mining [39]. It ranks the influence of features by measuring its occurrence in individual samples as well as in the entire dataset, and tends to select samples while rarely occur in others. The general equation is:

(1) $$TF-iDF_{i,j}=\frac{n_{i,j}}{\sum_{k}\;n_{k,j}}\log\frac{\left|D\right|}{\left|\left\{j:t_{i}\in d_{j}\right\}\right|}$$

Where *n*_*i,j*_ is the occurrence of the *i*-th feature, *t*_*i*_, in sample *d*_*j*_,$\left|\left\{j:t_{i}\in d_{j}\right\}\right|$is the total number of samples that contain feature *t*_*i*_, and |*D*| is the total number of samples in the dataset.

***mRMR*** (minimum-redundancy maximum-relevancy) is a mutual information-based feature selection method that selects a subset of features that correlates strongest to the classification categories with the least redundancy [40]. The criteria to be met for maximum relevance and minimum redundancy respectively are:

(2) $$\max D\left(S,c\right),D=\frac{1}{\left|S\right|}\sum_{x_{i}\in S}I\left(x_{i};c\right)$$

(3) $$\min R\left(S\right),R=\frac{1}{{\left|S\right|}^{2}}\sum_{x_{i}x_{j}\in S}I\left(x_{i};x_{j}\right)$$

Where *I* (*x;y*) is the mutual information between set *x* and *y*, *S* is the selected subset of features, *x*_*i*_ and *x*_*j*_ are different features in the subset and *c* is the sample state in question. Implementation of this method was downloaded from the Peng Lab website (http://penglab.janelia.org/proj/mRMR/), with the default discretization threshold 1.

***LEfSe*** (linear discriminant analysis effect size) is a recently published algorithm for high-dimensional biomarker discovery that identifies genomic features (genes, pathways, taxa) characterizing the differences between two or more biological conditions [41]. We benchmarked this feature selection method to see how well it predicted the age groups for new metagenomes using the same training and testing framework.

Both the TF-iDF and mRMR methods rank features in order of a probabilistic score, and select the top N features, where N is free variable determined by a predefined need for a certain number of features [42]. To examine the most probabilistically relevant features, we arbitrarily chose the top 10 differentiable features selected by TF-iDF and mRMR for the following analysis and show their discriminative power along age.

### Support vector machine (SVM)

We used a linear-kernel support vector machine to classify newly observed metagenomic samples to a particular group of samples. Since the abundances of functional signatures in metagenomic sample are often sparse (that is, certain functions seem to be sample-specific rather than universal) and therefore cannot be modeled by a particular distribution [40], SVM is robust and appropriate as a non-probabilistic binary linear classifier for the data.

To examine the classification for novel metagenomic samples, a subset was randomly drawn from the dataset, and a leave-one-out cross-validation was conducted to predict age. In each leave-one-out iteration, signature features were selected based on the abundances of functional features of the training, and supervised classification using SVM was performed on the left-out sample. The prediction accuracy and the area under receiver operating characteristic (ROC) curve (AUC) were measured. This process is repeated multiple times on different sub-datasets to show the mean and confidence interval of these measurements.

### Grubb’s test and support vector machine

One characteristic of TF-iDF is that it tends to pick features with less frequent occurrences. To determine if solely choosing low-occurrence features yields good classification performance, Grubb's test for detecting outliers is used for feature selection on the Qin *et al.* dataset. Similar to TF-iDF, Grubb’s test does not take into account the class information of samples. Since the features chosen by Grubb’s test do not improve classifier performance (as we show in the Results section), we therefore validate that TF-iDF is successfully distinguishing features that are significantly identifying particular classes of metagenomes, as opposed to solely choosing outliers. Similar to previous experiments, the functional signatures selected were Pfams that best classified the outlying metagenomes using SVM in a leave-one-out fashion. For each Pfam set in training that marked a sample as an outlier, SVM was performed to test the metagenome based on these Pfams, and the Pfams were assigned weight 1 if the classification was accurate. The total weight was computed and Pfams with the top 10 highest weights were then used for testing the left-out sample, and both accuracy and AUC were recorded.

### Transformation-based principal component analysis (tbPCA)

**Figure 1:**
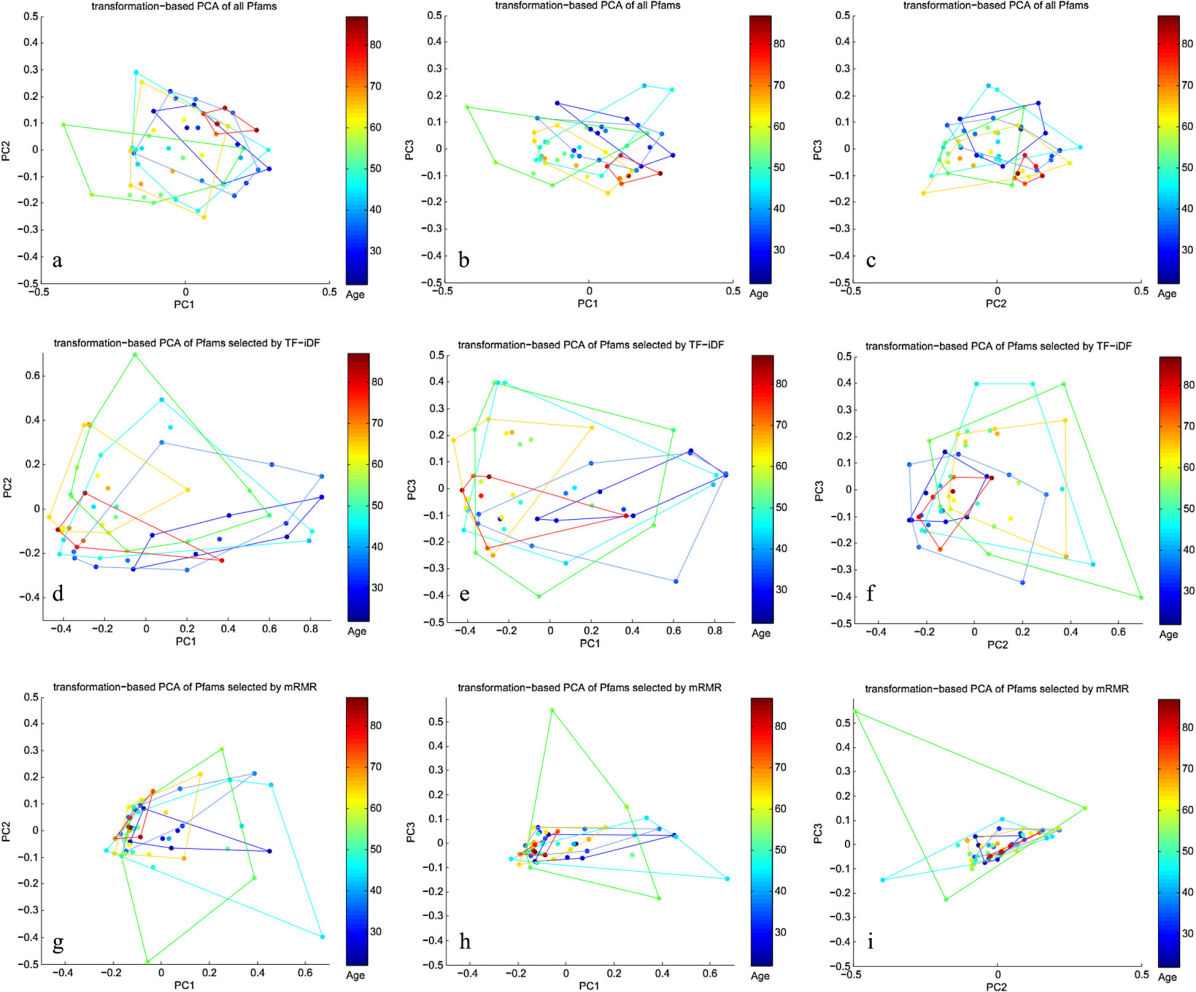
**Transformation-based PCA of age groups using Pfams.** Transformation-based PCA of age groups using the abundance of Pfams in the age-balanced dataset. Each color marks one age decade, except for red which corresponds to samples 70 to 90 years old. The first row used all Pfams present in each samples, the second and third rows used only top selected Pfams at age cutoff 40 by TF-iDF and mRMR separately. The columns are PC1 vs. PC2, PC1 vs. PC3 and PC2 vs. PC3 respectively. There is great overlap between most age groups, which explains why classification may be difficult. Besides the differences induced by source country of the metagenomes, mRMR reduces the variation within groups while TF-iDF facilitates linear classification. mRMR, minimum-redundancy maximum-relevancy; PCA, principal component analysis; Pfam, protein family; TF-iDF, term frequency-inverse document frequency.

Transformation-based PCA, described by Legendre and Gallagher, was performed to show the ordination of the samples in different age groups [43]. To construct the plots in Figure 1, the abundances of all and feature-selected Pfams were used, and the samples were colored by the corresponding age groups.

## Results

### Functional annotation

The proportion of the non-redundant sequences being assigned to different functional reservoirs as well as the phylogenetic assignment estimated by Qin *et al.* in [3] is shown in Table 1. We can see that compared to phylogenetic assignment, fewer sequences were annotated through the functional databases, but six times more feature categories were assigned, demonstrating that the resolving power of phylotypes is limited. One exception was the KEGG pathway, which was only able to annotate 18.7% of the sequences and yield 238 different features (that is, pathways). Loss of information due to the number of sequences that cannot be functionally annotated and large number of various functional descriptions have made it very difficult to identify the functions that are driving the differentiation between samples.

**Table 1:** Sequences being annotated by various functional reservoirs

**Samples**	**% of sequences annotated**	**Functional feature type**	**Number of unique features**
Qin *et al.* dataset	50.2%	Pfam	6343
	18.7%	KEGG pathway	238
	47.0%	KEGG Ontology	6015
	77.1%	Phylotype	~1150 species
Age-balanced dataset	52.0%	Pfam	5809
	47.5%	Gene Ontology	7079

Fewer sequences can be annotated with functional features compared to phylogenetic taxon, but with more categories

### Developing cutoffs for age classification

Both TF-iDF and mRMR have been recently shown to boost performance of taxonomic read classification [44]. We used linear-kernel SVM for classification, based on the selected Pfams picked by these two methods, in order to examine their ability of predicting the age (young vs. old) for new metagenomic samples. Unlike some physiologies that can be easily identified as a finite set of states, such as IBD diagnosis, BMI or diet-type, age is sampled yearly across a lifespan and the definition of young vs. old is ambiguous. We develop a series of cutoffs to separate the metagenomes as ‘young’ (defined as below or equal to the arbitrary cutoff) or ‘old’ (defined as above the arbitrary cutoff), sampled at the finest interval for the given dataset (with an interval of one year for the mid-ages that show to be more differentiable than young adults or seniors) to show how the prediction performance gradually changes in the process of host aging.

### Ordination of samples in different age groups

By applying tbPCA from the Methods section, we generated the ordination of samples of different age groups in the age-balanced dataset (Figure 1). Each color marks one age decade, except for the red which corresponds to samples 70 to 90 years old. Interestingly, without feature selection (Figure 1a-c), the oldest samples (six samples marked in red) are more tightly clustered compared to other age groups. This is most likely because these samples are all from Italy while the other age groups are mixtures of multiple countries, showing nationality as a source of variation between gut metagenomes [9].

However, aging trends can still be observed. With our ‘feature selection + SVM classifier’ framework, we optimized feature selection to discriminate between two age groups. For this example, we ran the selection to optimize under-40 and over-40 discrimination. Figure 1d-f demonstrate the effect of TF-iDF-selected features on the ordination. Compared with using all Pfams, using TF-iDF-selected functional signatures helps to widen the differences between age groups in proper order, and to make younger and older groups more linearly separable in the hyperspace regardless of the variation in each age decade, especially samples in their 20s (three from Spain, two from Japan and one from American) compared with samples in their 60s (three from Spain, three from France and four from Denmark). In the TF-iDF-selected ordination, a gradual shift from the right to left occurs as the host age increases. Similarly, Biagi *et al*. have shown how centenarians are differentiable from younger adults (25 to 40 years old) while the elderly (63 to 76 years old) overlapped with both young and centenarians, when using 16 rRNA data in their redundancy analysis [14]. This is similar to what we observed here when samples were better separated around mid-ages (Figure 1d-f). On the contrary, we can also see that the mRMR-selected signatures (Figure 1g-i) have made the age groups cluster more tightly and it is almost impossible to draw a straight line to distinguish between the young and old samples.

### Age classification using Qin *et al.* dataset

In our framework, TF-iDF and mRMR are used in combination with SVM, and leave-one-out cross-validation is performed on the Qin *et al.* dataset in 20 runs with 110 random samples in each subset.

**Figure 2:**
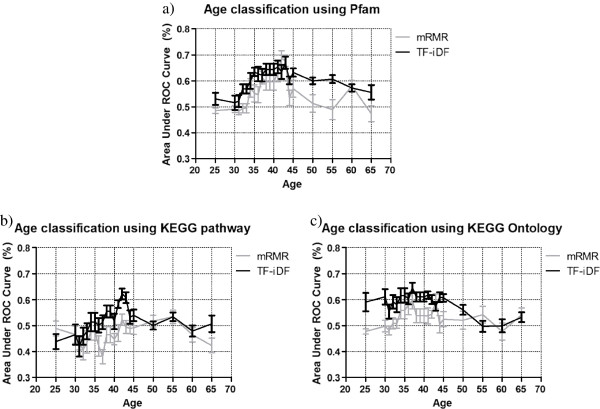
**Age detection on Qin *et al. *dataset using Pfam, KEGG pathway and KEGG Ontology.** Age detection using **(a)** Pfam **(b)** KEGG pathway and **(c)** KEGG Ontology on Qin *et al.* dataset show that classification performance increases towards mid-age cutoffs. TF-iDF appears to be a more accurate and consistent feature selection method for age detection compared to mRMR. KEGG, Kyoto Encyclopedia of Genes and Genomes; mRMR, minimum-redundancy maximum-relevancy; Pfam, protein family; TF-iDF, term frequency-inverse document frequency.

As is shown in Figure 2a, the classification performance gradually increases from age 30 to 43 and decreases after 43. This is true for both TF-iDF and mRMR, while the former outperforms the latter with higher average AUCs and smaller error bars (which shows the 95% confidence interval based on 20 independent runs). The highest AUCs occur within the range of 38 to 43 years old, where TF-iDF yields average AUCs of around 65% (the highest AUC 67.04 ± 2.35% occurs at age 43) and mRMR yields average AUCs >60% (with 67.78 ± 2.17 at age 43), indicating the ability of both feature selection methods to predict younger populations from older populations.

We also examined the age prediction using the KEGG Pathway and KEGG Ontology abundances (Figure 2b and c). For the KEGG pathways, although mRMR hovers around the 50% AUC chance line with wider error bars than TF-iDF, and the prediction gradually increases between age 38 to 42 with average AUCs generally over 55% (the highest AUC 62.22 ± 2.07 occurs at age 42), and decreasing thereafter. For the KEGG ontologies, we see that mRMR only has overall prediction performances over 50% AUC (the lower end of the error bar) around age 34 to 43, while TF-iDF yields AUCs around 60% from age 25 to 45 and decreasing thereafter with the error bars more narrow around the mid-ages. For the mid-ages, there is approximately 60% AUC even when taking into account the confidence interval, indicating that the occurrences of the few selected KEGG pathways have similarly good age prediction potential as the few selected Pfams described above. Both Pfam and KEGG signatures show that the best differentiation performance takes place at around the mid-ages of 38 to 43, suggesting that these alternations of human gut microbial functions may begin at an early age.

### Verification for TF-iDF findings using Qin *et al.* dataset

In most cases, we find that TF-iDF outperforms mRMR in age prediction. Different from mRMR, which looks for the least redundant subset of functional signatures to make the age prediction, TF-iDF is a feature selection method that does not take group information into consideration but chooses signatures that best characterize each sample against the rest. Concerned by this preference towards ‘rare’ features, we decided to use a method that would only choose outliers, Grubb’s test, to determine if solely choosing rare features would yield performance as good as TF-iDF. We assessed the AUCs with TF-iDF using Pfams at age 43 (where TF-iDF generates AUCs of 67.04 ± 2.35%), and the ‘Grubb’s test + SVM classification’ experiment generates AUCs of 50.48 ± 2.07% in 20 runs. Thus, the feature selection pattern may not be the only reason why TF-iDF works better. In fact, while TF-iDF aims to select signatures that have high frequency of occurrence in few metagenomes but low frequency of existence across all the metagenomes, there is a trade-off between such criterion and the random-subset experiment we designed, since signatures with too low of a frequency may not appear in many subsets, and thus cannot be consistently picked. Thus, TF-iDF-selected functional signatures in our age classification framework are those that appear in only a few individuals among the entire population at large frequencies. We hypothesize that the differences observed over age may appear to be induced by ‘accessory genes’ (genes that do not occur in all metagenomes but do occur in a subset of them [45]).

### Age classification using an age-balanced dataset

**Figure 3:**
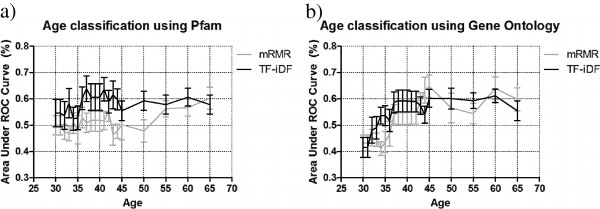
**Age detection on the age-balanced dataset using Pfam and Gene Ontology.** Age detection on the age-balanced dataset using **(a)** Pfam and **(b)** Gene Ontology shows that classification performance increases from younger age cutoffs towards mid-ages. Pfam, protein family.

Due to the bias of Qin *et al.* dataset towards the 40- to 60-year-old age range, we created a more uniformly distributed dataset along age (see Methods) to test our feature selection framework. We conducted similar leave-one-out cross-validation in 20 runs with 40 random samples in each subset. In Figure 3a, we show the age detection using Pfam signatures selected from the age-balanced dataset, where the AUCs again get slightly higher measurements round the mid-ages of 36 to 43 years old (>60% on average) for TF-iDF, yet the error bars are wide than when using the other dataset. This is most likely because we are using datasets over multiple geographical regions, and that the ‘accessory Pfams’ selected by TF-iDF may not be as consistent in a heterogeneous population. Still, mRMR performs generally 10% lower in AUCs than TF-iDF until 55-year-old divisions. We also tried Gene Ontology (GO) terms in our feature selection process. In Figure 3b, again both methods show a gradual increase from younger cutoffs to the mid-age cutoffs, with considerably good performance around 45 years old (60.20 ± 3.34% for TF-iDF and 64.74 ± 4.32 for mRMR).

**Figure 4:**
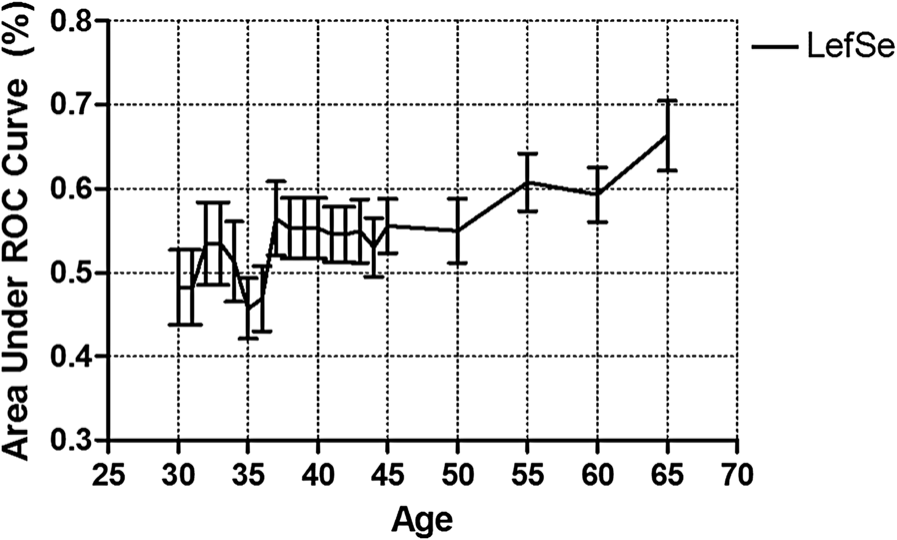
**Age detection using Pfam selected by LEfSe.** Age detection using Pfam selected by LEfSe on the age-balanced dataset shows that LEfSe identified features that predict age best at age 65, while marginally discriminating those above and below the mid-age range. Pfam, protein family; LEfSe, linear discriminant analysis effect size.

To verify that ‘feature selection + SVM classifier’ improved performance for age group separation, we compared TF-iDF and mRMR against a recently published feature identification algorithm used for metagenomes, linear discriminant analysis effect size (LEfSe). Figure 4 shows the results for age detection using LEfSe-selected Pfams combined with SVM classifier on the age-balanced dataset, in which leave-one-out cross-validation was performed with the same setting we used for TF-iDF and mRMR. We can see that the prediction performance reveals similar patterns to that of TF-iDF and mRMR around the mid-ages showing approximately 5% higher AUCs than younger cutoffs. However, LEfSe yields approximately 65% AUC for the 65-year-old case.

## Discussion

### Supervised classification for age

Through a simple ordination of protein families present in each metagenome’s unique gene set, we demonstrate that metagenomes across different age groups are largely variable and greatly overlapped, since individuals age at different rates. Nonetheless, through an optimization example of feature selection for classifying individuals under and over 40 years old, we show that mRMR tightly clusters the samples while TF-iDF enables better linear separation of the age groups. This is likely why TF-iDF performs better with the linear-kernel SVM classification we implemented.

By training and testing the SVM classifier on Pfams selected via TF-iDF and mRMR, we show that we can better classify the age (young vs. old) of novel metagenomic samples as the age cutoffs move gradually from younger to mid-ages (around 38 to 43) with AUCs 10 to 15% higher than chance and with stable confidence intervals, on both Qin *et al.* dataset and age-balanced dataset. Similar improvement of AUCs around mid-ages was observed when running our method on KEGG pathways, KEGG ontologies and Gene Ontologies. Specifically, classification was based on only a few features selected by TF-iDF and mRMR, indicating that even abundances of very limited number of identified functional signatures are representative of host physiology variations. We observe that TF-iDF works better than mRMR for most cases, most likely due to the fact that TF-iDF selects features that best classify the metagenomes with a linear classifier while mRMR identifies a subset of signatures that tightly clusters the samples but makes it harder to classify. Experimenting on random subsets and leave-one-out cross-validation also prevent TF-iDF from simply choosing the most rare features by chance, as revealed by comparing to the Grubb’s test.

We also conduct an experiment using the same framework on the age-balanced dataset using a recently published method LEfSe for feature selection, which identified some age-related Pfams that yielded better prediction at age 65.

We wish to remark on the variation of the classification performance. We note that 15% of the Pfams annotated were unique to only one metagenome. Either these are truly ‘accessory’ Pfams or the microbiome gene content is under-sampled even though the Qin *et al.* dataset is the most comprehensive gut microbiome dataset to date. While we are able to see trends in the age differentiation using functional signatures, we are still far from understanding which protein families or pathways play a major role in aging. Also, the problem is compounded by the fact that a functional feature can be dependent on multiple host physiologies. In fact, we see a large area of future research to be focused on decoupling multiple factors that affect metagenomes, in order to gain insight into a single physiology.

### Biological interpretation

By comparing results from Table 1 and Figure 2, we notice that the overall prediction performance positively correlates with the number of sequences that can be annotated. Therefore, the characterization of new metagenomes based on gene content should be reliant on more sequences being functionally interpretable.

While the age-balanced dataset turns out to be more complicated for mRMR, it shows the effectiveness of feature selection on age-differentiable signatures similar to that of TF-iDF on Qin *et al.* dataset. One Pfam that is consistently selected by mRMR when using the Qin *et al.* dataset (Additional file 1: Table S2) is CbiN (PF02553), which is involved in the biosynthesis of vitamin B12. Analysis on Qin *et al.* dataset shows that this Pfam has the largest difference in presence among all the Pfams we are studying, existing in 55% of the young (using age cutoff of 43) and only 11% of the old, which is consistent with the observation that vitamin B12 deficiency is often reported in the elderly [46]. It is also notable that some of the top Pfams selected by mRMR yielding good prediction involve domains of unknown functions, indicating that finer interpretation of sequence functions is required to gain more insights into what roles they may serve in the process of aging.

As mentioned in the above discussion, TF-iDF tends to pick the functional signatures that are ‘accessory’. Yet the term ‘accessory’ here has a different meaning from features that only appear in one or two individuals, but features that may be present in a small group of individuals as opposed to the entire population. We also notice that our age detection method does not work as well on the age-balanced dataset as on the Qin *et al.* dataset, which is probably due to the inherent complexity within the dataset: the age-balanced dataset drew samples from six different countries, thus contained more variation within each age group. Yet still, we find that TF-iDF assigns highest credits to some similar Pfams when experimenting on both datasets, indicating a consistent differentiating power of these Pfams in characterizing metagenome age over different datasets. Additional file 1: Tables S2 and Table S3 list the selected Pfams for Qin *et al.* and the age-balanced dataset at age 43, which presents better prediction (in regards of average AUC and confidence interval) for both datasets. One top Pfam selected by TF-iDF in both datasets, for example, is the hemolysin-type calcium-binding repeat (PF00353), which plays a role in the lytic activity of exotoxins such as hemolysin, cyclolysin and leukotoxin [47]. In Qin *et al.* dataset, it is recognized more in the older samples (47%) than in the younger samples (30%), implying a higher possibility of microbial toxin secretion in the old.

Additional file 1: Table S4 shows the Pfams selected by LEfSe at age cutoff 65, which outperforms other age cutoffs for this feature selection method. The most significant Pfams are involved in DNA damage (PF00533) [48], bacterial DNA replication (PF00521) [49] and ribosomal proteins (PF00542). LEfSe also identified glycosyl transferase family 2 (PF00521) which transfers sugar from UDP-glucose, UDP-N-acetylgalactosamine, GDP-mannose or CDP-abequose to a range of substrates including cellulose, dolichol phosphate and teichoic acids, consistent with the finding that glycosyltransferase (COG0438) correlates with age [4].

KEGG-based classification indicates multiple metabolic and biosynthesis pathways altered with age (Additional file 1: Table S5). Arachidonic acid metabolism is an age-related human pathway involved in lipid peroxidation [50]. We also discovered reduced biosynthesis function related to cell adhesion and cell surface carbohydrates, such as mucin-type O-glycans, which are known to be decreased in some prostate and colorectal cancer types [51]. We also identified additional KEGG pathways that are associated with altered proteins in various cancer types, such as prostate and bladder cancer. Furthermore, while the data suggests that bacterial degradation systems related to the ubiquitin-mediated proteolysis pathway is impaired, which is ATP-dependent, autophagy is gained. In summary, these changes reflecting a switch from anabolism to catabolism contribute to age-related mechanisms described in prokaryotes [52], which may be conserved in human pathways.

Additionally, we found that GO terms classify samples better for age cutoffs over 45, for both TF-iDF and mRMR. Additional file 1: Table S6 shows the list of GO terms selected at this age cutoff. While mRMR does not select functional signatures as consistently as TF-iDF, they both highlight some of the age-relevant GO terms. Downregulated activity of some reductases in the old, for example, is identified by TF-iDF, including glycine reductase (GO0030699) and dimethyl sulfoxide reductase (GO0009389). Stress responses and immune system compromise are also observed in the old, such as cellular response to sulfate starvation (GO0009970), blood vessel remodeling (GO0001974), phagocytosis (GO0006909) and evasion by virus of host immune response (GO0030683). The activity of dextransucrase (GO0047849), an enzyme belongs to the family of glycosyltransferases, decreased in the old, which is consistent with what LEfSe identified.

## Conclusions

Age has been studied as a potential characteristic influencing the constitution and activity of gut microbiomes. Here we aim to identify various functional signatures that differentiate metagenomes over a broad range of age, in extension of previous studies on age-related phylogenetic differences in metagenomes and altered functional compositions at extreme ages. We show that by optimizing feature selection for supervised classification, age-relevant functional signatures can be discovered, including DNA damage, vitamin synthesis and various metabolic functions. While ‘age classification’ based on the metagenome is far from being realized due to undersampling in the population and known functional annotations, our approach indicates that metagenomic samples can be differentiated by age and identifies potential healthy or unhealthy age-relevant metabolic attributes in the human microbiome.

## Abbreviations

AUC: area under ROC curve; BLAST: Basic Local Alignment Search Tool; COGs: Clusters of Orthologous Groups of proteins; GA: gathering threshold; GO: Gene Ontology; IBD: inflammatory bowel disease; KEGG: Kyoto Encyclopedia of Genes and Genomes; LEfSe: linear discriminant analysis effect size; mRMR: minimum-redundancy maximum-relevancy; PCA: principal component analysis; Pfam: protein family; ROC: receiver operating characteristic; SVM: support vector machine; tbPCA: transformation-based principal component analysis; TF-iDF: term frequency-inverse document frequency; UniProt: Universal Protein Resource databases.

## Competing interests

The authors declare that they have no competing interests.

## Authors’ contributions

YL and GR conceived the research study and designed the methods and experiments, YL implemented the methods and conducted all experiments and data analysis, YL drafted the manuscript, and YL, AK, and GR participated in the interpretation of the results. All authors have contributed to, read, and approved the final manuscript.

## Supplementary Material

Additional file 1 **Table S1.** Details of the demographics of age-balanced dataset (sorted by age). **Table S2.** Top selected Pfams on Qin *et al*. dataset (age cutoff is 43). **Selection rate is the number of times one Pfam is picked over the total number of permutations trained on different random subsets. *Top Pfams that are selected by both the Qin *et al.* and the age-balanced dataset. **Table S3.** Top selected Pfams on an age-balanced dataset (age cutoff is 43). *Top Pfams that are selected by both the Qin *et al.* and the age-balanced dataset. **Table S4.** Top selected Pfams by LEfSe on an age-balanced dataset (age cutoff is 65). **Table S5.** Top selected KEGG pathways on Qin *et al*. dataset (age cutoff is 42). **Table S6.** Top selected GO terms on an age-balanced dataset (age cutoff is 45). Click here for file
