# Supplementary material for: Selecting age-related functional characteristics in the human gut microbiome
Source: Microbiome. 2013 Jan 9;1:2. doi: 10.1186/2049-2618-1-2 (PMC3869192; doi:10.1186/2049-2618-1-2)
Supplement: Additional file 1 — Table S1. Details of the demographics of age-balanced dataset (sorted by age). Table S2. Top selected Pfams on Qin et al. dataset (age cutoff is 43). **Selection rate is the number of times one Pfam is picked over the total number of permutations trained on different random subsets. *Top Pfams that are selected by both the Qin et al. and the age-balanced dataset. Table S3. Top selected Pfams on an age-balanced dataset (age cutoff is 43). *Top Pfams that are selected by both the Qin et al. and the age-balanced dataset. Table S4. Top selected Pfams by LEfSe on an age-balanced dataset (age cutoff is 65). Table S5. Top selected KEGG pathways on Qin et al. dataset (age cutoff is 42). Table S6. Top selected GO terms on an age-balanced dataset (age cutoff is 45). [file 2049-2618-1-2-S1.doc]

*Table S1.* Details of the demographics of age-balanced dataset (sorted by age)

| Sample ID | Age | Nationality | BMI | IBD |
| --- | --- | --- | --- | --- |
| V1UC8 | 22 | Spain | 25.4 | N |
| InR | 24 | Japan |  | N |
| V1CD1 | 25 | Spain | 17.9 | Y |
| V1UC15 | 25 | Spain | 22.8 | Y |
| F1T | 28 | Japan |  | N |
| Subject7 | 28 | America |  | N |
| F1S | 30 | Japan |  | N |
| V1UC9 | 32 | Spain | 30.4 | N |
| O2UC11 | 34 | Spain | 18.7 | Y |
| V1CD15 | 34 | Spain | 19.0 | Y |
| InD | 35 | Japan |  | N |
| F2W | 36 | Japan |  | N |
| V1CD6 | 36 | Spain | 18.5 | Y |
| F2V | 37 | Japan |  | N |
| Subject8 | 37 | America |  | N |
| V1UC6 | 38 | Spain | 23.2 | N |
| V1CD12 | 41 | Spain | 20.2 | Y |
| O2UC12 | 43 | Spain | 21.6 | Y |
| MH19 | 44 | Denmark | 20.0 | N |
| MH37 | 44 | Denmark | 24.0 | N |
| InA | 45 | Japan |  | N |
| V1UC10 | 45 | Spain | 27.3 | Y |
| O2UC18 | 48 | Spain | 25.7 | Y |
| MH50 | 49 | Denmark | 25.1 | N |
| MH54 | 49 | Denmark | 20.3 | N |
| MH77 | 49 | Denmark | 24.9 | N |
| O2UC20 | 51 | Spain | 24.0 | Y |
| V1UC13 | 51 | Spain | 28.5 | Y |
| V1UC14 | 53 | Spain | 20.3 | Y |
| MH38 | 54 | Denmark | 22.0 | N |
| MH56 | 54 | Denmark | 25.4 | N |
| MH64 | 54 | Denmark | 23.2 | N |
| MH68 | 54 | Denmark | 29.0 | N |
| O2UC24 | 55 | Spain | 28.8 | Y |
| O2UC17 | 56 | Spain | 21.9 | Y |
| MH39 | 58 | Denmark | 23.1 | N |
| NO4 | 60 | France | 23.8 | N |
| NO3 | 61 | France | 22.0 | N |
| V1UC21 | 62 | Spain | 25.2 | Y |
| NO1 | 63 | France | 23.1 | N |
| V1UC18 | 63 | Spain | 28.7 | N |
| MH17 | 64 | Denmark | 21.8 | N |
| MH44 | 64 | Denmark | 24.5 | N |
| MH79 | 64 | Denmark | 20.0 | N |
| MH40 | 67 | Denmark | 20.9 | N |
| O2UC13 | 68 | Spain | 23.4 | Y |
| E | 70 | Italy |  |  |
| G | 72 | Italy |  |  |
| C | 77 | Italy |  |  |
| D | 80 | Italy |  |  |
| A | 84 | Italy |  |  |
| B | 87 | Italy |  |  |

*Table S2.* Top selected Pfams on Qin’s dataset (age cutoff is 43)

** Selection Rate is the number of times one Pfam is picked over the total number of permutations trained on different random subsets.

* Top Pfams that are selected by both the Qin *et al.*’s and the age-balanced dataset.

|  | Pfam Accession | Pfam Name | Selection Rate ** |
| --- | --- | --- | --- |
| TF-iDF | PF00353.13 | HemolysinCabind* | 1.00 |
|  | PF00415.12 | RCC1* | 1.00 |
|  | PF01473.14 | CW_binding_1* | 1.00 |
|  | PF02493.14 | MORN* | 1.00 |
|  | PF05594.8 | Fil_haemagg* | 1.00 |
|  | PF07538.5 | ChW* | 1.00 |
|  | PF05017.8 | TMP* | 0.99 |
|  | PF07661.7 | MORN_2 | 0.93 |
|  | PF12789.1 | PTR | 0.83 |
|  | PF00400.26 | WD40 | 0.48 |
|  | PF01469.12 | Pentapeptide_2* | 0.34 |
|  | PF05658.8 | Hep_Hag | 0.18 |
| mRMR | PF08406.4 | CbbQ_C | 1.00 |
|  | PF11653.2 | VirionAssem_T7 | 0.83 |
|  | PF02553.9 | CbiN | 0.80 |
|  | PF05876.6 | Terminase_GpA | 0.78 |
|  | PF11794.2 | HpaB_N | 0.69 |
|  | PF03669.7 | UPF0139 | 0.64 |
|  | PF06117.5 | DUF957 | 0.44 |
|  | PF07287.5 | DUF1446 | 0.42 |
|  | PF01974.1 | tRNA_int_endo | 0.38 |
|  | PF10326.3 | 7TM_GPCR_Str | 0.32 |
|  | PF03241.7 | HpaB | 0.24 |
|  | PF08617.4 | CGI-121 | 0.21 |
|  | PF06300.6 | Tsp45I | 0.18 |
|  | PF09754.3 | PAC2 | 0.17 |
|  | PF03537.7 | DUF297 | 0.17 |

*Table S3.* Top selected Pfams on an age-balanced dataset (age cutoff is 43)

* Top Pfams that are selected by both the Qin *et al.*’s and the age-balanced dataset.

|  | Pfam Accession | Pfam Name | Selection Rate |
| --- | --- | --- | --- |
| TF-iDF | PF00353.13 | HemolysinCabind* | 1.00 |
|  | PF00419.14 | Fimbrial | 0.99 |
|  | PF07538.5 | ChW* | 0.95 |
|  | PF07980.5 | SusD | 0.92 |
|  | PF01469.12 | Pentapeptide_2* | 0.73 |
|  | PF02493.14 | MORN* | 0.73 |
|  | PF05594.8 | Fil_haemagg* | 0.73 |
|  | PF03382.8 | DUF285 | 0.62 |
|  | PF07676.6 | PD40 | 0.62 |
|  | PF01473.14 | CW_binding_1* | 0.57 |
|  | PF05017.8 | TMP* | 0.56 |
|  | PF00415.12 | RCC1* | 0.34 |
|  | PF13004.1 | BACON | 0.34 |
|  | PF00577.14 | Usher | 0.25 |
|  | PF07460.5 | NUMOD3 | 0.21 |
|  | PF01345.12 | DUF11 | 0.19 |
| mRMR | PF04883.6 | DUF646 | 0.90 |
|  | PF12314.2 | IMCp | 0.32 |
|  | PF06130.6 | PduL | 0.31 |
|  | PF09652.4 | Cas_VVA1548 | 0.30 |
|  | PF04286.6 | DUF445 | 0.26 |
|  | PF02920.9 | Integrase_DNA | 0.20 |
|  | PF08821.5 | CGGC | 0.19 |
|  | PF12083.2 | DUF3560 | 0.18 |
|  | PF01862.10 | PvlArgDC | 0.17 |
|  | PF09234.4 | DUF1963 | 0.16 |
|  | PF10711.3 | DUF2513 | 0.15 |

*Table S4.* Top selected Pfams by *LEfSe* on an age-balanced dataset (age cutoff is 65)

|  | Pfam Accession | Pfam Name | Selection Rate |
| --- | --- | --- | --- |
| *LEfSe* | PF00533.20 | BRCT | 0.77 |
|  | PF00542.13 | Ribosomal_L12 | 0.67 |
|  | PF00535.20 | Glycos_transf_2 | 0.62 |
|  | PF00521.1 | DNA_topoisoIV | 0.47 |
|  | PF00437.14 | GSPII_E | 0.45 |
|  | PF00529.14 | HlyD | 0.41 |
|  | PF00441.1 | Acyl-CoA_dh_1 | 0.34 |
|  | PF02684.9 | LpxB | 0.27 |
|  | PF00528.16 | BPD_transp_1 | 0.26 |
|  | PF00070.21 | Pyr_redox | 0.25 |
|  | PF00436.19 | SSB | 0.24 |
|  | PF01182.1 | Glucosamine_iso | 0.22 |
|  | PF00438.14 | S-AdoMet_synt_N | 0.20 |
|  | PF00532.15 | Peripla_BP_1 | 0.20 |
|  | PF03840.8 | SecG | 0.19 |
|  | PF02682.10 | AHS1 | 0.17 |
|  | PF00563.14 | EAL | 0.16 |

*Table S5.* Top selected KEGG pathways on Qin’s dataset (age cutoff is 42)

|  | KEGG ID | KEGG Pathway | Selection Rate | % In Young | % In Old |
| --- | --- | --- | --- | --- | --- |
| TF-iDF | map00512 | Mucin type O-Glycan biosynthesis | 1.00 | 68.42% | 62.86% |
|  | map00522 | Biosynthesis of 12-, 14- and 16-membered macrolides | 1.00 | 26.32% | 23.81% |
|  | map00590 | Arachidonic acid metabolism | 1.00 | 89.47% | 77.14% |
|  | map00633 | Nitrotoluene degradation | 1.00 | 94.74% | 87.62% |
|  | map04120 | Ubiquitin mediated proteolysis | 0.99 | 78.95% | 61.90% |
|  | map05012 | Parkinson's disease | 0.89 | 52.63% | 47.62% |
|  | map04510 | Focal adhesion | 0.76 | 31.58% | 34.29% |
|  | map04810 | Regulation of actin cytoskeleton | 0.67 | 68.42% | 65.71% |
|  | map05215 | Prostate cancer | 0.62 | 47.37% | 43.81% |
|  | map04020 | Calcium signaling pathway | 0.57 | 21.05% | 31.43% |
|  | map00540 | Lipopolysaccharide biosynthesis | 0.54 | 100.00% | 98.10% |
|  | map00072 | Synthesis and degradation of ketone bodies | 0.32 | 100.00% | 79.05% |
|  | map00906 | Carotenoid biosynthesis | 0.19 | 36.84% | 41.90% |
|  | map05219 | Bladder cancer | 0.16 | 52.63% | 45.71% |
|  | map00253 | Tetracycline biosynthesis | 0.15 | 94.74% | 88.57% |
| mRMR | map01056 | Biosynthesis of type II polyketide backbone | 0.99 | 0.00% | 8.57% |
|  | map04080 | Neuroactive ligand-receptor interaction | 0.81 | 0.00% | 6.67% |
|  | map00901 | Indole alkaloid biosynthesis | 0.71 | 26.32% | 8.57% |
|  | map02040 | Flagellar assembly | 0.66 | 63.16% | 60.00% |
|  | map05213 | Endometrial cancer | 0.64 | 21.05% | 4.76% |
|  | map04120 | Ubiquitin mediated proteolysis | 0.58 | 78.95% | 61.90% |
|  | map05012 | Parkinson's disease | 0.44 | 52.63% | 47.62% |
|  | map00253 | Tetracycline biosynthesis | 0.43 | 94.74% | 88.57% |
|  | map04140 | Regulation of autophagy | 0.38 | 0.00% | 5.71% |
|  | map05211 | Renal cell carcinoma | 0.34 | 84.21% | 68.57% |
|  | map00601 | Glycosphingolipid biosynthesis - lacto and neolacto series | 0.31 | 57.89% | 44.76% |
|  | map04614 | Renin-angiotensin system | 0.27 | 21.05% | 10.48% |
|  | map05110 | Vibrio cholerae infection | 0.26 | 31.58% | 20.95% |
|  | map00351 | DDT degradation | 0.26 | 94.74% | 86.67% |
|  | map05215 | Prostate cancer | 0.23 | 47.37% | 43.81% |
|  | map04340 | Hedgehog signaling pathway | 0.21 | 5.26% | 14.29% |
|  | map04940 | Type I diabetes mellitus | 0.21 | 47.37% | 43.81% |
|  | map04740 | Olfactory transduction | 0.20 | 5.26% | 11.43% |
|  | map05221 | Acute myeloid leukemia | 0.16 | 21.05% | 7.62% |
|  | map04020 | Calcium signaling pathway | 0.16 | 21.05% | 31.43% |

*Table S6.* Top selected GO terms on an age-balanced dataset (age cutoff is 45)

|  | GO term ID | Gene Ontology Name | Selection Rate | % In Young | % In Old |
| --- | --- | --- | --- | --- | --- |
| TF-iDF | GO:0030699 | glycine reductase activity | 0.97 | 86% | 53% |
|  | GO:0043064 | flagellum organization | 0.94 | 91% | 77% |
|  | GO:0015833 | peptide transport | 0.91 | 95% | 100% |
|  | GO:0009425 | bacterial-type flagellum basal body | 0.89 | 91% | 83% |
|  | GO:0008137 | NADH dehydrogenase (ubiquinone) activity | 0.81 | 95% | 97% |
|  | GO:0047849 | dextransucrase activity | 0.71 | 68% | 47% |
|  | GO:0009389 | dimethyl sulfoxide reductase activity | 0.65 | 73% | 63% |
|  | GO:0005274 | allantoin uptake transmembrane transporter activity | 0.64 | 50% | 27% |
|  | GO:0016163 | nitrogenase activity | 0.58 | 82% | 60% |
|  | GO:0016151 | nickel cation binding | 0.51 | 95% | 90% |
|  | GO:0008556 | potassium-transporting ATPase activity | 0.50 | 95% | 80% |
|  | GO:0003746 | translation elongation factor activity | 0.37 | 95% | 100% |
|  | GO:0008982 | protein-N(PI)-phosphohistidine-sugar phosphotransferase activity | 0.20 | 95% | 100% |
|  | GO:0015716 | phosphonate transport | 0.20 | 73% | 47% |
|  | GO:0009296 | flagellum assembly | 0.19 | 86% | 90% |
|  | GO:0008863 | formate dehydrogenase (NAD+) activity | 0.17 | 86% | 80% |
| mRMR | GO:0009970 | cellular response to sulfate starvation | 0.90 | 0% | 47% |
|  | GO:0001974 | blood vessel remodeling | 0.76 | 0% | 37% |
|  | GO:0006909 | phagocytosis | 0.42 | 0% | 30% |
|  | GO:0008979 | prophage integrase activity | 0.31 | 50% | 43% |
|  | GO:0030683 | evasion by virus of host immune response | 0.23 | 5% | 27% |
|  | GO:0019903 | protein phosphatase binding | 0.21 | 32% | 7% |
|  | GO:0004446 | inositol-hexakisphosphate phosphatase activity | 0.20 | 5% | 33% |
|  | GO:0002009 | morphogenesis of an epithelium | 0.20 | 0% | 27% |
|  | GO:0000164 | protein phosphatase type 1 complex | 0.18 | 23% | 0% |
|  | GO:0004797 | thymidine kinase activity | 0.18 | 100% | 90% |
|  | GO:0030335 | positive regulation of cell migration | 0.18 | 5% | 27% |
|  | GO:0043281 | regulation of cysteine-type endopeptidase activity involved in apoptotic process | 0.16 | 5% | 30% |
